# Human Stem Cell Models of SARS-CoV-2 Infection in the Cardiovascular System

**DOI:** 10.1007/s12015-021-10229-4

**Published:** 2021-08-08

**Authors:** Kyle Ernzen, Aaron J. Trask, Mark E. Peeples, Vidu Garg, Ming-Tao Zhao

**Affiliations:** 1 Center for Cardiovascular Research, The Abigail Wexner Research Institute, Nationwide Children’s Hospital, Columbus, OH USA; 2 The Heart Center, Nationwide Children’s Hospital, Columbus, OH USA; 3 MCDB Graduate Program, The Ohio State University, Columbus, OH USA; 4 Department of Pediatrics, The Ohio State University College of Medicine, Columbus, OH USA; 5 Center for Vaccine and Immunity, The Abigail Wexner Research Institute, Nationwide Children’s Hospital, Columbus, OH USA; 6 Department of Physiology and Cell Biology, The Ohio State University College of Medicine, Columbus, OH USA

**Keywords:** SARS-CoV-2, COVID-19, Heart failure, Human pluripotent stem cells, Cytokine storm

## Abstract

The virus responsible for coronavirus disease 2019 (COVID-19), severe acute respiratory syndrome coronavirus 2 (SARS-CoV-2), has infected over 190 million people to date, causing a global pandemic. SARS-CoV-2 relies on binding of its spike glycoprotein to angiotensin-converting enzyme 2 (ACE2) for infection. In addition to fever, cough, and shortness of breath, severe cases of SARS-CoV-2 infection may result in the rapid overproduction of pro-inflammatory cytokines. This overactive immune response is known as a cytokine storm, which leads to several serious clinical manifestations such as acute respiratory distress syndrome and myocardial injury. Cardiovascular disorders such as acute coronary syndrome (ACS) and heart failure not only enhance disease progression at the onset of infection, but also arise in hospitalized patients with COVID-19. Tissue-specific differentiated cells and organoids derived from human pluripotent stem cells (hPSCs) serve as an excellent model to address how SARS-CoV-2 damages the lungs and the heart. In this review, we summarize the molecular basis of SARS-CoV-2 infection and the current clinical perspectives of the bidirectional relationship between the cardiovascular system and viral progression. Furthermore, we also address the utility of hPSCs as a dynamic model for SARS-CoV-2 research and clinical translation.

**Graphical abstract:**

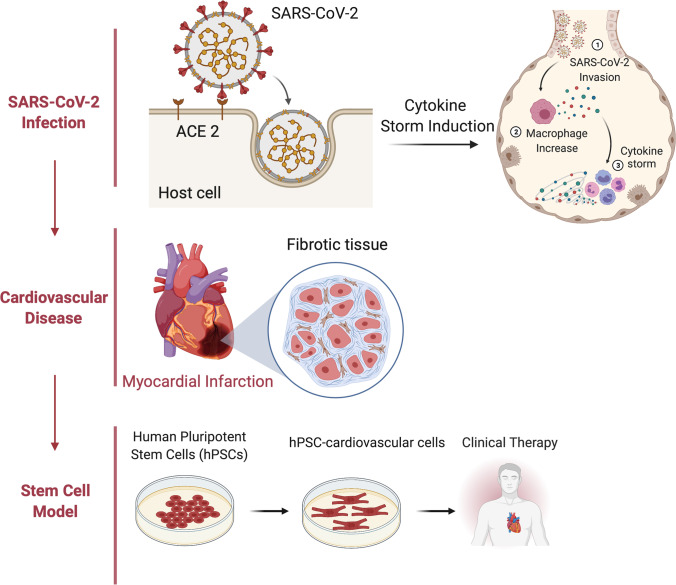

## Introduction

Coronavirus disease 2019, caused by severe acute respiratory syndrome coronavirus 2, was first reported in Wuhan, China in December 2019 [1, 2]. As of July 2021, more than 190 million cases of COVID-19 have been reported globally, contributing to over 4 million deaths [3]. The rapid spread of this virus over the course of one year is due to the high transmissivity of SARS-CoV-2 by viral aerosols that can persist in the air for hours [4]. Inhalation of these aerosol particles may quickly lead to infection, resulting in a wide spectrum of symptoms that vary from a minor headache to life-threating pneumonia [2]. To counter the high efficiency of viral transmission, governments worldwide have imposed strict quarantine protocols and travel bans that have negatively impacted the job security of millions of people [5]. Currently, 15 vaccines have been approved globally, with 56 vaccine candidates in development [6]. The center for disease control and prevention (CDC) indicates that global administration of effective vaccines could take months to years to achieve herd immunity. In addition, vaccinated individuals still have the capacity to contract and spread the virus. Therefore, continued development of new disease models and potential therapeutic regimens are clearly warranted. Due to the ever-changing landscape of the COVID-19 pandemic, the parallel development of curative treatments alongside vaccines continues to be increasingly relevant and vital. For example, the recently discovered Delta variant of SARS-CoV-2 has demonstrated increased pathogenicity and reduced sensitivity to monoclonal antibodies in vaccine recipients, necessitating that models for curative treatments continue to be thoroughly investigated [7].

SARS-CoV-2 belongs to the beta coronavirus subfamily of positive sense RNA viruses, as do SARS-CoV and Middle East respiratory syndrome coronavirus (MERS-CoV). SARS-CoV and SARS-CoV-2 cause similar pathogenicity since they share the same spike (S) protein and use similar cellular proteases for viral entry. Like all coronaviruses, their virion membranes include a large S glycoprotein that protrudes from the surface of the virion and gives these viruses their corona-like appearance in electron micrographs. This crown-like morphology of the S protein is a distinct feature that gives the coronavirus a suitable title [8]. The S protein enables viral entry into the host cell’s cytoplasm through recognition and binding to its host cell membrane receptor, ACE2, for SARS-CoV and SARS-CoV-2 and dipeptidyl peptidase-4 (DPP4) for MERS-CoV [9, 10]. The S protein is dependent upon two distinct cleavage steps for proper function: a cleavage near the protein’s midpoint by a furin-like enzyme to divide the protein into two separate subunits and a cleavage of the subsequent S2 subunit by transmembrane protease serine 2 (TMPRSS2), which is critical for activating the fusion potential of the virus [11, 12]. This second cleavage is a key contributing factor as to why the SARS-CoV-2 pandemic has been so much more robust and rapid than the previous SARS-CoV and MERS-CoV outbreaks.

SARS-CoV-2 infection can lead to a wide range of symptoms including shortness of breath, fever, sore throat, cough, muscle pain, and loss of taste and/or smell. While these symptoms can be fatal for some, others may be completely asymptomatic [13]. Furthermore, there is increasing evidence that SARS-CoV-2 infection may cause serious long-term side effects for both hospitalized and asymptomatic individuals, such as anxiety, depression, cognitive impairment, and dyspnea [14, 15]. The unpredictable and long-term impact of COVID-19 on the health of infected patients highlights the dire need for curative treatments and models of study. Individuals with high risk for severe SARS-CoV-2 infection typically have comorbidities such as diabetes, obesity, cardiovascular disease, hypertension, chronic obstructive pulmonary disease (COPD), liver disease, and renal disease [16, 17]. An examination of these comorbidities reveal that a wide range of organ systems are involved with enhanced disease progression, including the cardiovascular and respiratory systems, liver, and kidneys. This is due to the respective host cell receptor ACE2 being expressed in many of these systems. In fact, aberrant expression of ACE2 is quite common in diseases of the heart, liver, lungs, and kidneys [18]. Despite the presence of ACE2, numerous autopsy reports have revealed that SARS-CoV-2 is rarely detected in the cardiomyocytes of deceased COVID-19 patients, indicating the limited capacity of the virus to infect these cells directly in vivo [19, 20]. Given that cardiovascular complications frequently arise in COVID-19 patients, these reports demonstrate how the virus may compromise organ systems through both direct and indirect modes of action.

Severe SARS-CoV-2 infection is common in patients with comorbidities, especially individuals with respiratory and cardiovascular diseases. For example, respiratory diseases such as COPD present at the onset of infection often lead to an increased mortality rate in hospitalized patients [21]. SARS-CoV-2 also has the capacity to induce novel COPD or ARDS in infected patients, which typically arise from the severe pneumonia and debilitated immunity caused by the infection [17, 22]. Additionally, cardiovascular disease has the potential to enhance patient mortality as an established comorbidity upon initial infection or as a newly developed symptom caused by viral infection [17, 23–26]. In fact, COVID-19 may lead to an array of cardiovascular disorders such as myocardial injury, arrhythmias, ACS, venous thromboembolism (VTE) and heart failure [27]. While development of cardiovascular disease is commonly a long-term effect of COVID-19 that may persist months after recovery, some patients do not survive the infection due to the severity and rapid induction of these respective disorders [25, 27].

In this review, we summarize the molecular basis of SARS-CoV-2 infection, including the viral entry mechanisms that lead to induction of the cytokine storm. We outline the multiple clinical presentations of cardiovascular damage and the bidirectional consequences of COVID-19. Finally, we discuss the efficacy and therapeutic potential of utilizing human stem cell-based models for studying SARS-CoV-2 infection.

## Molecular Basis of SARS-CoV-2 Infection

**Fig. 1:**
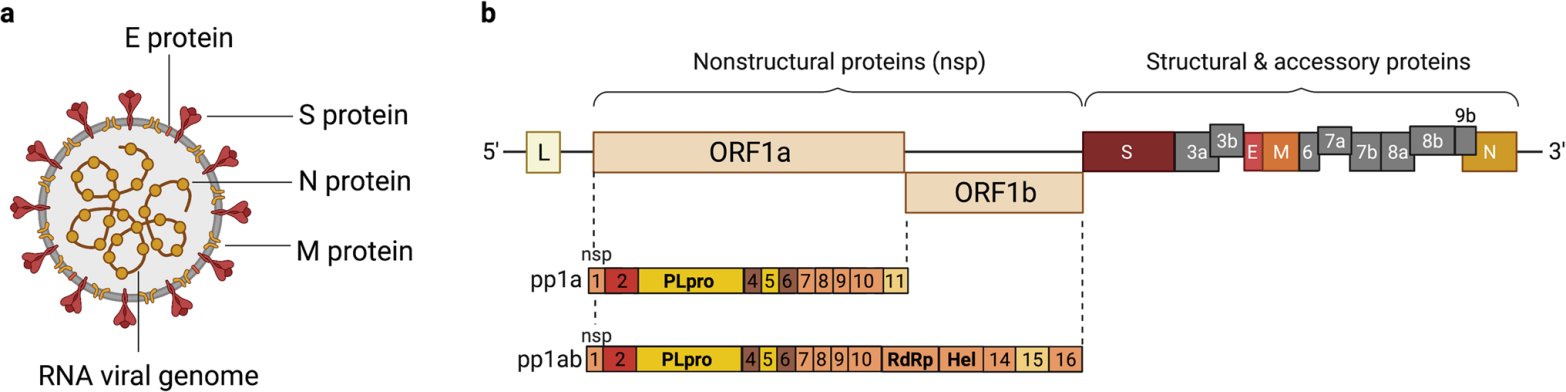
The structure and genome of the SARS-CoV-2 virus. **a** The SARS-CoV-2 virion particle consists of the spike (S), envelope (E), nucleocapsid (N), and membrane (M) structural proteins that encase the single-stranded RNA (ssRNA) viral genome. These four proteins play key roles in the processes of viral entry, assembly, and replication. The S protein is required for receptor binding and membrane fusion with the host cell. Once inside the host cell, the N protein serves to package and protect the RNA viral genome into helical ribonucleoprotein complexes. During the viral replication process, E proteins travel to the endoplasmic reticulum and Golgi to assist with virion assembly and budding. Lastly, the M protein functions as an essential mediator for virion assembly and interacts with the S, N, and E proteins to ensure viral retention, stabilization, and envelope formation. **b** Two-thirds of the SARS-CoV-2 genome at the 5’ terminal end encodes the pp1a and pp1ab polyproteins, which are subsequently cleaved into 16 nonstructural proteins responsible for the replicase complex. The remaining one-third of the genome at the 3’ terminal region encodes the four structural proteins of the virus. The illustration was created with BioRender.com

The coronavirus genome consists of 14 open reading frames (ORFs), with two-thirds of the genome at the 5’ terminal encoding the pp1a and pp1ab polyproteins. These critical polyproteins are subsequently cleaved into 16 nonstructural proteins that are essential for the functional composition of the replicase complex [28, 29]. The remainder of the genome near the 3’ terminal region encodes four viral structural proteins known as the spike (S), envelope (E), nucleocapsid (N) and membrane (M) glycoproteins (Fig. 1). Responsible for host cell receptor binding and membrane fusion, the S1 and S2 domains of the spike protein are critical [11, 29, 30]. Each of the three S1 domains contains a receptor-binding domain (RBD) capable of interacting with ACE2 [18]. The RBD has two different conformational states: the lying state (inaccessible conformation) and the exposed state (accessible conformation), in which one or more of these RBDs can be exposed at a time [31]. Due to the presence of a novel solvent-exposed furin like cleavage site and the S protein’s 10 to 20-fold greater affinity for ACE2, SARS-CoV-2 has posed a substantially greater threat than SARS-CoV and MERS-CoV [12, 32, 33].

**Fig. 2:**
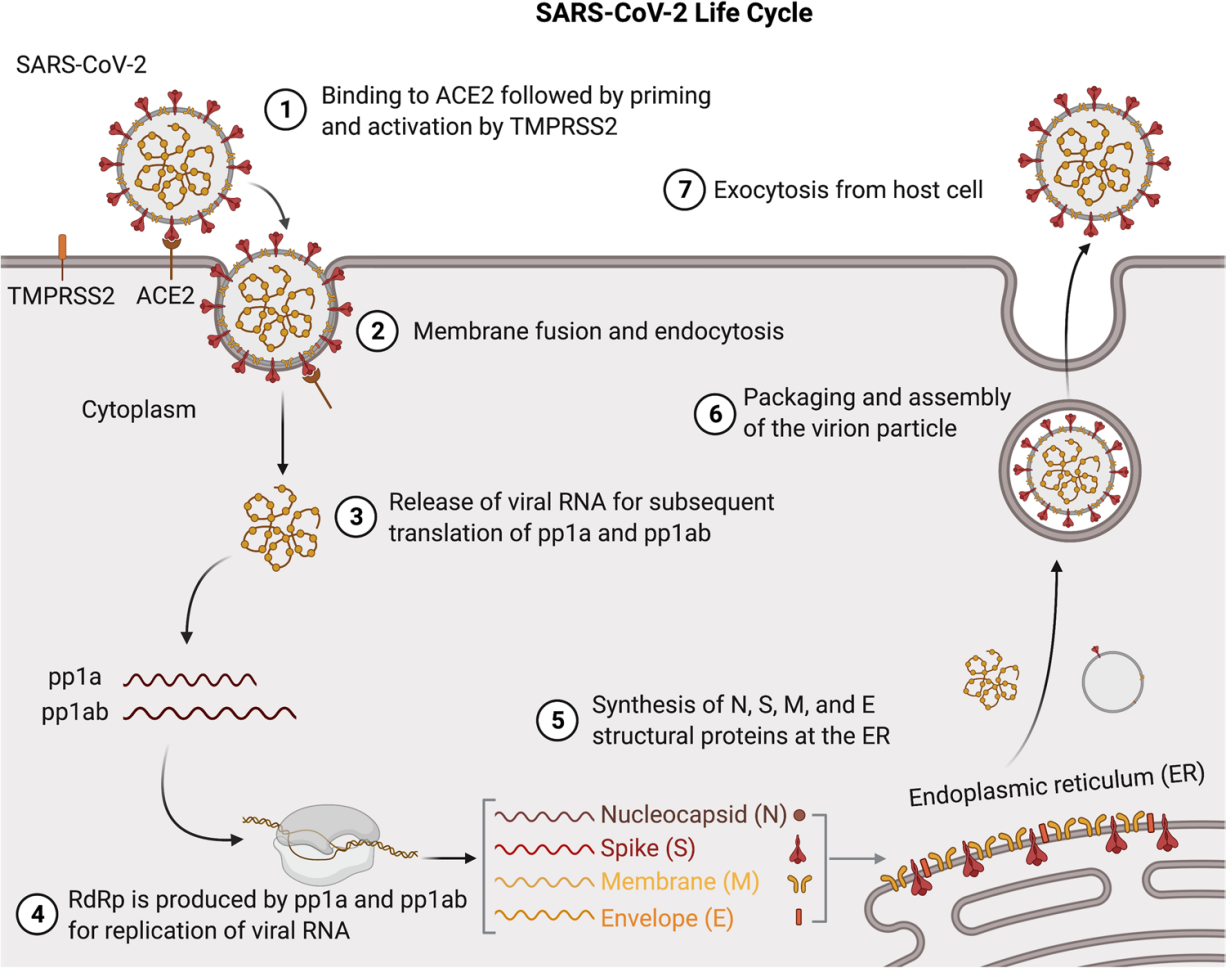
The life cycle of the SARS-CoV-2 virus. (1) Cellular entry of SARS-CoV-2 is mediated by binding of the S protein to the host cell’s ACE2 receptor. (2) TMPRSS2 is then responsible for cleavage at the S1/S2 boundary site (“priming”) and the S2 site (“activation”) which facilitates membrane fusion and endocytosis into the host cell. (3) Upon release into the cytoplasm, the first ORF of the viral ssRNA is translated into the pp1a and pp1ab polyproteins. (4) These polyproteins are subsequently processed into non-structural proteins (nsps) that compose the RNA-dependent RNA polymerases (RdRps), which serve to replicate the viral RNA. (5) The viral RNA subsequently becomes translated at the ER and Golgi complex for synthesis of the S, E, N, and M structural proteins. (6) Genomic ssRNA and structural proteins are then packaged into new viral particles, with the M protein functioning as the primary organizer for virion assembly. (7) Once the assembly process is complete, virions will exit the infected host cell through exocytosis. The illustration was created with BioRender.com

Once the S protein is bound to a host cell’s ACE2 receptor, viral entry is dependent upon a necessary protease “activation” step (Fig. 2). “Activation” involves cleavage of S2 by TMPRSS2 N-terminal to the fusion peptide, leading to a fusion-competent state and induction of membrane fusion [34, 35]. Although this is the most common mechanism for cellular entry of SARS-CoV-2, viral particles may alternatively enter through an endosomal pathway mediated by cysteine proteases cathepsin B or cathepsin L [34, 36]. This second method of SARS-CoV-2 entry into a target cell has been shown to occur in immortalized cells that express ACE2 but not TMPRSS2 on their plasma membrane. This situation has been studied primarily in two cultured cell lines: Vero, derived from African green monkey kidney which expresses that primate’s ACE2 protein, and HEK293 cells stably transfected to express ACE2. Both of these cell lines support SARS-CoV-2 infection and replication and have been used to generate virus stocks for in vitro experiments. In these cells, SARS-CoV-2 virions bind to ACE2, are endocytosed, and reach the late endosome or lysosome where cathepsin L or B cleaves the S2 subunit at the same site TMPRSS2 would have. Once cleaved, the remaining transmembrane portion of the S2 subunit causes fusion between the virion membrane and the lysosomal membrane, releasing the genome into the cytoplasm to initiate infection [37].

The endosomal pathway of SARS-CoV-2 infection is particularly relevant for viral entry into cardiomyocytes. Although *ACE2* is expressed in the heart, the amount of ACE2 protein expressed across individual cardiomyocytes is highly variable [18, 38]. Furthermore, the expression levels of TMPRSS2 are extremely low throughout the heart, while the levels of the cathepsin L and furin are higher [39]. A study by Bailey et al. assessed the mechanisms of SARS-CoV-2 infection in a cardiac-like environment by introducing the virus to 3-D tissues consisting of human PSC-derived cardiomyocytes (PSC-CMs), macrophages, and fibroblasts. Their findings revealed that only cardiomyocytes were positive for SARS-CoV-2, with electron microscopy demonstrating the presence of virion particles specifically within perinuclear endosomal-like structures. They confirmed the endosomal pathway of viral entry by testing the cathepsin inhibitor, E64, and the TMPRSS2 inhibitor, camostat mesylate, in infected human PSC-CMs. E64 eliminated the presence of SARS-CoV-2 in these cardiomyocytes whereas camostat mesylate had no effect [38]. Similarly, a report by Bojkova et al. confirmed these findings through a 3-D cardiosphere tissue model, in which they demonstrated the ability of cathepsin inhibitor N-Acetyl-L-leucyl-L-leucyl-L-methional (ALLM) to decrease the level of S protein expression in human PSC-CMs [40]. Although these in vitro studies indicate an endosomal route of entry for SARS-CoV-2 specifically in cardiomyocytes, it is important to note that SARS-CoV-2 is rarely detected in the cardiomyocytes of autopsy patients [19, 20].

This endosomal route of viral entry is not the main route for SARS-CoV-2 infection. SARS-CoV-2 isolated from a patient contains the furin site in its S protein. The virus infects Vero cells inefficiently, but as it is passaged it grows to higher titers. The S protein of these viruses have mutations that disrupt the furin cleavage site. Therefore, these viruses can no longer infect human airway cells [41, 42]. Conceptually, a similar selection could occur in vivo with the originally infecting virus containing the furin site, enabling it to infect airway cells, but produce a virus with a mutated furin site that would infect and spread in cells expressing ACE2 but not TMPRSS2.

Once the virus has entered the cell, the first ORF of the viral genomic RNA can immediately be translated into the pp1a and pp1ab polyproteins which are subsequently cleaved into the components of the RNA-dependent RNA polymerases (RdRps) and proteins that restructure the cytoplasmic membrane vesicles for use in replication [29]. The RNA polymerase then replicates the viral RNA and the subgenomic RNAs for the genes at the 3’ end of the genome. The mRNAs encoding the S, E, N, and M virion structural proteins are translated on the ER membranes and accumulate in the ER-Golgi-intermediate (ERGIC) compartment between the ER and the Golgi. Genomic copies associate with the N protein and then with the ERGIC where the virion is formed by budding into the ERGIC. The virion is moved through the Golgi where the S protein is cleaved into S1 and S2 by a furin-like protease, and the virions are released by exocytosis from the host cell, initiating a new round of infection by the SARS-CoV-2 virions [29, 43] (Fig. 2).

Upon SARS-CoV-2 infection, the immune system will initially identify the foreign viral antigens. This leads to antigen-presenting cells processing these viral antigens for subsequent recognition by natural killer and CD8-positive cytotoxic T cells. These cells will then induce both innate and adaptive immunity responses, leading to a series of signaling pathways that activate several pro-inflammatory cytokines such as interleukin-6 (IL-6), interleukin-1 (IL-1), and tumor necrosis factor alpha (TNF-α) [44, 45]. Severe cases of COVID-19 often develop when these pro-inflammatory cytokines are rapidly overproduced, leading to a “cytokine storm” that may lead to tissue damage, organ failure, and even death [44]. As SARS-CoV-2 primarily affects the respiratory system, acute lung injury and development of ARDS are common consequences of this cytokine hyper-stimulation [46]. Cytokine storm may also contribute to the development of severe cardiovascular disorders such as myocardial injury, acute myocardial infarction (AMI), VTE, and heart failure [27, 47, 48]. Early intervention to either prevent or mitigate this deadly inflammatory response has been widely regarded as a key factor in promoting favorable patient outcomes [46, 49].

As the primary receptor for cellular entry of SARS-CoV-2, ACE2 is widely expressed in the lungs, heart, liver, kidneys, and intestines [50, 51]. Proper equilibrium of ACE and ACE2 is critical for maintenance of the renin–angiotensin–aldosterone system (RAAS), in which both of these enzymes work together to regulate many physiological processes, including but not limited to blood pressure, electrolyte homeostasis, and inflammation responses [52]. RAAS homeostasis and ACE2 expression are also affected by gender, age, diet, and disease. For example, elderly men are at a greater risk for severe SARS-CoV-2 infection due to high expression of ACE2 in the lungs. Studies have also shown that ACE2 activity is increased for individuals who consume too much salt, glucose, and fats [53]. RAAS dysregulation and aberrant expression of ACE2 is especially common in diseases such as COPD, heart disease, chronic kidney disease, and diabetes. COVID-19 patients with any one of these comorbidities are at a significantly higher risk for severe infection and enhanced disease progression [18, 53].

## Clinical Perspectives-Cardiovascular Disease

The physiological impact of SARS-CoV-2 is not limited to the respiratory system; the widespread expression of both the ACE2 receptor and TMPRSS2 throughout organ systems such as the heart, liver, lungs, and kidneys could potentially allow for a significant degree of viral tropism [18, 54]. The effects of SARS-CoV-2 on the cardiovascular system are of particular interest due to the bidirectional relationship between cardiovascular disease and COVID-19 on patient mortality rates [27]. While the precise mechanisms are not entirely clear on how cardiovascular disease leads to a more severe prognosis in COVID-19 patients, proposed methods include: inability of the cardiovascular system to accommodate high viral load alongside a reduction in systemic oxygenation, electrolyte variance causing additional stress on the compromised heart, and a weakened immune system lacking effective T cell signaling [55]. As proposed by Libby and Lüscher [56], COVID-19 may be an “endothelial disease” associated with net increased inflammation, oxidative stress, thrombosis, impaired barrier function, vasoconstriction, and myocarditis.

SARS-CoV-2 infection has the potential to induce myocardial injury through non-ischemic mechanisms such as hypoxic respiratory infection, myocarditis, pulmonary thrombosis, and cardiac adrenergic overproduction from cytokine storm [57] (Table 1). Elevated levels of cardiac troponin I is a key indicator of myocardial injury. In addition, patients with myocardial injury often exhibit an increase in inflammatory biomarkers such as IL-6 and C-reactive protein (CRP). Patients with myocardial injury also display electrocardiographic abnormalities and echocardiographic irregularities in the left and right ventricles of the heart [58]. Data from multiple clinical studies show the positive correlation between myocardial injury and increased patient mortality. One study in New York City consisted of 2,736 COVID-19 patients, 985 (36%) of which presented with increased concentrations of troponin, indicative of myocardial injury. This study revealed that patients with a low (e.g., troponin I > 0.03 to 0.09 ng/ml; n ¼ 455; 16.6%) or high (e.g., troponin I > 0.09 ng/dl; n ¼ 530; 19.4%) degree of myocardial injury had significantly greater mortality rates (p < 0.001) [59]. In a cohort study of 416 patients diagnosed with COVID-19 in Wuhan, China, 82 patients (19.7%) experienced cardiac injury while being hospitalized. The mortality rates of these patients were significantly higher than patients without cardiac injury (42 of 82 [51.2%] vs 15 of 334 [4.5%]; *P* < 0.001) [25]. Another study from Wuhan, China assessed 112 COVID-19 patients, with the majority demonstrating normal troponin levels upon admission. Troponin levels were observed to rise in 42 patients (37.5%) throughout hospitalization, with significant increase in 14 patients (12.5%) who ultimately died [60]. Furthermore, a retrospective study of 54 COVID-19 patients from Wuhan, China found that 24 patients (44.4%) developed myocardial injury throughout their stay in the hospital. In comparison to patients without myocardial injury, they demonstrated an increased mortality rate (14 (60.9%) vs. 8 (25.8%), *P* = 0.013) and higher levels of C-reactive protein (153.6 ng/L vs. 49.8 ng/L, *P* < 0.01) [61]. Overall, these findings illustrate how frequently myocardial injury can be developed from SARS-CoV-2 infection and lead to more dire outcomes.

**Table 1:** Mortality rates for COVID-19 patients with cardiovascular disease

CVD	Symptoms	Mortality R=rates with COVID-19 and CVD	References
Myocardial Injury	Increased levels of Troponin T and CRP	24% (n = 223) 51% (n = 82) 61% (n = 23)	Lala et al. [59] Deng et al. [60] He et al. [116]**
Acute Myocardial Infarction	ST segment elevation Increased D-dimer levels	72% (n = 18) 39% (n = 28)	Bangalore et al. [23] Stefanini et al. [64]
Venous Thromboembolism	Pulmonary embolism Alveolar damage	58% (n = 12)	Wichmann et al. [26]
Heart Failure	Chronic inflammation Cardiac fibrosis	57% (n = 21) 55% (n = 117) 64% (n = 28)	Inciardi et al. [65] Paranjpe et al. [73]* Zhou et al. [74]

*CVD*, Cardiovascular disease; *CRP*, C-reactive protein

*Article is a preprint and has not yet been peer reviewed

**Article is in Chinese whereas the Abstract is in English

ACS, or more specifically, AMI, shares the hallmark COVID-19 symptom of chest pain. Clinical presentation of AMI varies between intermittent chest pain or persistent pain that continues for several days [62]. Potential pathways in which SARS-CoV-2 causes AMI include plaque rupture, microthrombi, and coronary spasms caused by widespread inflammation and cytokine overproduction [27]. Although there is an array of biomarkers that can be utilized for the diagnosis of ACS, ST segment elevation and increased D-dimer levels are frequently used to detect ACS in COVID-19 patients [23, 63, 64]. A retrospective study performed in Strasbourg, France focused on 106 ACS patients that were admitted in the hospital. Although only 7 of these patients were diagnosed with COVID-19, a greater occurrence of type 2 myocardial infarction (29% vs. 4%, *P* = 0.0497) and elevated D-dimer levels (5,650 μg/l [interquartile range (IQR) 1,905–13,625 μg/l] vs. 400 μg/l [IQR 270–1,050 μg/l], *P* = 0.02) were detected in these patients [63]. These results demonstrate the capability of SARS-CoV-2 to potentially enhance the progression of ACS. In another study of 18 hospitalized COVID-19 patients with suspected AMI, 10 of the patients already presented with ST-segment elevation upon admittance, while the remaining 8 patients developed ST-segment elevation throughout their time in the hospital. Of these 18 patients, 13 (72%) of them did not survive during their stay [23]. Additionally, a retrospective study from Lombardy, Italy analyzed 28 COVID-19 patients with ST-elevation myocardial infarction, 24 (85.7%) of which demonstrated AMI as the first symptom prior to receiving a positive COVID-19 test result. The SARS-CoV-2 infection was ultimately fatal for 11 (39.3%) of these patients [64, 65]. In summation, these findings show the potential of COVID-19 to either enhance ACS progression or develop new ACS in infected patients.

Responsible for more than 100,000 deaths annually, VTE is the third most common acute cardiovascular disorder, which includes the disorders of both deep vein thrombosis and pulmonary embolism. Since VTE commonly causes respiratory distress and has a higher incidence rate in hospitalized patients, VTE has been frequently observed in patients with severe cases of COVID-19 [66, 67]. As a potential risk factor for VTE, COVID-19 stimulates the coagulation pathway and may cause extensive alveolar damage and inflammation [66]. Mechanisms for this coagulopathy are proposed to result from the COVID-19-induced cytokine storm, which is directly associated with increased inflammation, coagulation, and platelet induction [48]. Clinical data indicate both the increased incidence and mortality rate of VTE in COVID-19 patients [26, 68, 69]. A study from the Netherlands analyzed 184 intensive care unit (ICU) patients with COVID-19 from three separate Dutch hospitals. Of the 139 patients that remained in the ICU at the time of data collection, 37 patients (27%) were confirmed to have VTE, with 25 of them being diagnosed with pulmonary embolism [68]. Additionally, a cohort study of 198 hospitalized COVID-19 patients in Amsterdam, Netherlands focused specifically on VTE incidence for the 75 patients (38%) that required treatment in the ICU. A total of 39 patients (20%) were diagnosed with VTE, with the cumulative incidence rate substantially increasing over a 21-day period. It was also suggested that diagnosis of VTE was associated with fatal patient outcomes [69]. Furthermore, a prospective cohort study in Hamburg, Germany performed comprehensive virologic tests on 12 consecutive patients with deaths directly related to COVID-19. The virologic testing involved sample extraction from the heart, lungs, pharynx, kidney, saphenous vein and venous blood. They found that 7 (58%) of them had VTE. Pulmonary embolism was attributed as the main cause of death in 4 of these patients [26]. These studies demonstrate the relatively high incidence rate of VTE in COVID-19 patients, resulting in a serious risk factor that is associated with increased mortality.

Heart failure is characterized by the inability of the heart to pump an adequate supply of blood to sufficiently support the body’s needs. Heart failure typically involves chronic inflammation, in addition to the possibility of cardiac fibrosis, compromised cardiac tissue, loss of proper cardiac function and death [70]. Since COVID-19 is known to promote systemic inflammation and the overproduction of cytokines, SARS-CoV-2 infected patients are at a much higher risk for heart failure. This is especially relevant for severe cases of SARS-CoV-2, in which respiratory failure and a limited supply of oxygen places a tremendous burden on the myocardium [47]. In addition to inducing heart failure in COVID-19 patients, SARS-CoV-2 may also exacerbate disease progression in patients who have been previously diagnosed with chronic heart failure [71]. A meta-analysis of multiple COVID-19 publications associated with cardiovascular dysfunction reveals that patients with either pre-existing or newly developed heart failure are at a greater risk for poor patient outcomes including death [57, 72, 73]. A retrospective cohort study from Wuhan, China examined an array of different risk factors in 191 COVID-19 patients. A new onset of heart failure was observed in 44 patients, with 28 (64%) of them not surviving their stay in the hospital [74]. In another retrospective study, 113 deceased COVID-19 patients were characterized by their clinical complications and comorbidities. Of them, 41 (36%) of these patients experienced heart failure, which was more likely to develop in patients with cardiovascular comorbidities [24]. Together, the findings reveal the bidirectional relationship between COVID-19 and heart failure; both diseases are serious risk factors that pose a significant threat to mutual disease progression and patient outcomes.

## Human Stem Cell-Based Models

Common models used to study SARS-CoV-2 include immortalized cell lines and genetically modified mice [75, 76]. Although these models have demonstrated significant utility in studying the mechanisms of viral entry and replication, they fail to effectively mimic the physiological conditions of different tissue systems in humans [77]. Since SARS-CoV-2 infects multiple cell types across different tissues, hPSC models may serve as a good platform to comprehensively study SARS-CoV-2 infection. hPSCs, which encompass embryonic stem cells (ESCs) and induced pluripotent stem cells (iPSCs), have the capability to differentiate into a multitude of organ systems related to SARS-CoV-2 tropism, such as alveolar type 2 (AT2) cells in the lungs, cardiomyocytes, pancreatic cells, hepatocytes, kidney epithelial cells, and enterocytes [78]. Despite hPSC derivatives not being completely physiologically equivalent to these respective cell types in vivo, this model could serve as a competent in vitro substitute. Given that hPSCs can also be maintained almost indefinitely through laboratory cell culture, this versatile model serves as an excellent method to understand the multi-organ consequences of SARS-CoV-2 infection.

As the primary target for viral infection, the respiratory system is the focal point for studying SARS-CoV-2. Several groups have demonstrated the capability of hPSCs to effectively model SARS-CoV-2 infection in multiple organ systems [79–82] (Table 2). Han et al. established a lung organoid hPSC model to demonstrate the capability of these cells to be permissive to SARS-CoV-2 infection, activate a significant increase in chemokine transcripts, and be inhibited by US Food and Drug Administration (FDA)-approved drugs such as imatinib and mycophenolic acid [79]. Additionally, Samuel et al. employed hESC-derived lung organoids to overexpress ACE2 amongst a large variety of epithelial subtypes including AT2 cells. They tested a panel of anti-androgenic drugs against the COVID-19 infection and found a drastic decrease in the number of infected cells in the drug-treated group [82]. Similarly, Huang et al. showed that hPSC-derived AT2 cells induced a noticeable immune response upon SARS-CoV-2 infection. They also recapitulated the efficacy of remdesivir and a TMPRSS2 inhibitor, camostat mesylate, in blocking SARS-CoV-2 infection [80]. Stem cell models also have the potential to determine the relationship between relevant COVID-19 comorbidities and infection severity. Purkayastha et al. briefly exposed airway basal stem cells (ABSCs) to cigarette smoke and subsequently infected these cells with SARS-CoV-2. These cells displayed an increase in the number of infected cells accompanied by a reduction in the interferon response, ultimately resulting in a more severe disease phenotype [81]. Collectively, a large variety of differentiated hPSC subtypes can be applied to understand the cellular response against SARS-CoV-2 infection. This is also relevant for the cytokine storm response, which could potentially be measured through secretome analysis from different stem cell-derived organoid models infected with SARS-CoV-2. For example, the combined methods of liquid chromatography-mass spectrometry and antibody microarray analysis have demonstrated great promise for secretome characterization in models involving hPSC-derived cardiomyocytes (hPSC-CMs) [83].

**Table 2:** Human stem cells as a model for studying SARS-CoV-2 infection

Organs affected	Stem cell model	Outcomes	References
Lungs and colon	Lung organoids Colonic organoids	-Permissive to SARS-CoV-2 -Upregulated chemokine transcripts -Inhibited infection with FDA-approved drugs	Han et al. [79]
Lungs and heart	hESC-derived cardiac cells lung organoids	-Androgen signaling modulated ACE2 levels -Anti-androgenic drugs reduced ACE2 expression and protected hESC lung organoids against SARS-CoV-2 infection	Samuel et al. [82]
Lungs	iPSC-derived AT2 cells	-Upregulation of NF-kB signaling -Remdesivir and TMPRSS2 inhibitor demonstrated efficacy against infection	Huang et al. [80]
Lungs	human ABSCs	-Cigarette smoke exposure increased cellular infection and reduced interferon response -Interferon β-1 treatment abrogated viral infection	Purkayastha et al. [81]
Lungs	MSCs	-MSC transplantation improved pulmonary function and symptoms -Increase in peripheral lymphocytes and IL-10 -Decrease in CRP, TNF-α, and over-activated cytokine-secreting immune cells	Leng et al. [89]
Heart	hiPSC-CMs	-Permissive to infection, leading to apoptosis and cessation of beating -Stimulation of innate immune response and antiviral gene pathways -Inhibition of metabolic pathways and ACE2 expression	Sharma et al. [92]
Heart	hiPSC-CMs	-Upregulation in genes involving interferon signaling, apoptosis, and ROS -Remdesivir and cathepsin inhibitor ALLM blocked viral infection	Bojkova et al. [40]
Heart	hPSC-CMs	-Permissive to infection, leading to rapid cell death -Infection impaired electrophysiological and contractile properties	Marchiano et al. [96]
Heart	hiPSC-CMs	-ATR kinase inhibitor berzosertib demonstrated antiviral activity	Garcia et al. [95]
Heart	hPSC-CMs	-BRD inhibitor INCB05432 reduces ACE2 expression and SARS-Cov-2 infection in cardiac cells	Mills et al. [97]*

*hiPSC*, human induced pluripotent stem cell; *hESC*, human embryonic stem cell; *AT2*, alveolar epithelial type 2; *ABSC*, airway basal stem cell; *MSC*, mesenchymal stem cell; *IL-10*, interleukin 10; *CRP*, C-reactive protein; *TNF-α*, tumor necrosis factor alpha; *CM*, cardiomyocyte; *ROS*, reactive oxygen stress; *ALLM*, N-acetyl-L-leucyl-L-leucyl-L-methional; *ATR*, ataxia telangiectasia and Rad3-related; *BRD*, bromodomain

*Article is a preprint and has not yet been peer reviewed

Mesenchymal stem cells (MSCs) represent another stem cell model that has significant promise for studying the implications of SARS-CoV-2 infection and virus-induced respiratory damage due to their regenerative, immunomodulatory and antimicrobial properties [84, 85]. MSCs are easy to acquire and have low cytotoxicity in several clinical studies [84]. SARS-CoV-2 induction of the cytokine storm has been attributed to many severe complications in the lungs such as ARDS and COPD [17, 22]. Because MSCs decrease inflammation and attenuate a hyperactive immune response, pre-clinical studies have achieved considerable success in utilizing MSC-based transplantation therapy against ARDS and acute lung injury. These findings could potentially be applied to clinical studies targeting the respiratory manifestations upon severe SARS-CoV-2 infection [84–86]. MSCs reduce inflammation through induction of nuclear factor Nrf2 which serves as a critical transcription factor for activation of the anti-inflammatory response gene *heme oxygenase-1* (*HO-1*) [87]. Furthermore, basal expression of the ICOS ligand (ICOSL) by MSCs induces regulatory T-cells to suppress hyperinflammation and adverse immune responses [88]. Leng et al. determined the efficacy of MSC transplantation in seven patients with COVID-19 from Beijing, China. Following a 14-day assessment period, they observed favorable clinical outcomes: in the absence of any notable adverse effects, MSC transplantation greatly improved pulmonary function and overall symptom presentation by decreasing levels of C-reactive protein and hyper-stimulated cytokine-secreting immune cells. Gene expression studies revealed that none of the transplanted MSCs became infected with SARS-CoV-2 throughout the treatment [89]. A couple more studies [90, 91] have evaluated the safety and efficacy of infusion of umbilical cord MSCs in patients with severe COVID-19 and have shown promise for how MSCs can be used as a therapeutic approach to alleviate the severe respiratory symptoms of SARS-CoV-2 infection. However, these studies are not sufficient to support the clinical applications of MSCs in treating COVID-19, which is reflected by the recent NIH recommendation against the use of MSCs for the treatment of COVID-19 except in a clinical trial (https://www.covid19treatmentguidelines.nih.gov/therapies/cell-based-therapy/).

The prospect of modeling SARS-CoV-2 infection using hPSC-CMs has been extensively pursued by several groups. Sharma et al. used hPSC-CMs to examine the mechanisms of cardiomyocyte-specific SARS-CoV-2 infection. Their findings not only confirm that hPSC-CMs are permissive to SARS-CoV-2 infection, but also reveal that viral entry activates apoptosis and cessation of beating 72 h after infection. Viral infection induced an innate immune response in hPSC-CM model through upregulation of interleukin immunomodulators and antiviral pathway genes [92]. Furthermore, Bojkova et al. assessed the molecular response of hPSC-CMs to SARS-CoV-2 infection and the efficacy of anti-viral drugs in deterring viral progression. They discovered that SARS-CoV-2 infection of hPSC-CMs was dependent on the cathepsin-mediated endosomal pathway for viral entry. Their findings also demonstrated the ability of cathepsin inhibitor ALLM and viral RNA-dependent RNA polymerase inhibitor remdesivir to reduce S protein expression in hPSC-CMs [40].

Because hPSC-CMs serve as a relevant physiological model of the heart, these cells will be useful for understanding the mechanisms and therapeutic strategies associated with cardiovascular disease in the setting of COVID-19. Furthermore, while most other cell types can simply be obtained from tissue excisions, the only robust source for human cardiomyocytes is from hPSC [77]. A recent review thoroughly details how hPSC-CMs are employed for studying many cardiovascular complications such as fibrosis and ischemia-induced injury [93]. hPSC-CMs have also shown strong potential as model for assessing the cardiac safety of new drugs against SARS-CoV-2 [94]. Several reports have used hPSC-CMs to investigate SARS-CoV-2 infection in clinical perspectives [95–97]. Marchiano et al. indicated that SARS-CoV-2 infection changed the electrophysiological properties of hPSC-CMs by decreasing electrical conduction velocity and depolarization spike amplitude [96]. In order to assess the antiviral effects of a panel of protein kinase inhibitors, Garcia et al. screened these compounds and found that berzosertib, an ATR kinase inhibitor, showed considerable efficacy against SARS-CoV-2 in hPSC-CMs [95]. Lastly, Mills et al. utilized human PSC-derived cardiac organoids to test bromodomain protein (BRD) inhibitors against BRD4 which was an intracellular mediator of cardiac dysfunction from SARS-CoV-2 induced cytokine over-activation. An inhibitor, INCB054329, led to a four-fold reduction in ACE2 expression and a decrease in SARS-CoV-2 infection of cardiomyocytes [97]. These studies show great potential for how hPSC-derived cardiovascular cells can be utilized for understanding the cardiovascular implications of SARS-CoV-2 infection.

Despite the promise of hPSCs as a versatile model for COVID-19 study, there are several limitations. Characteristics of hPSCs such as immunogenic potential, epigenetic status, batch consistency, and maturation properties often vary between patients [98]. For example, heterogeneous mixtures of atrial, nodal, and ventricular subtypes are present in hPSC-CMs [99]. This variability of hPSC models poses complications for experimental procedures such as high throughput drug screening and cell replacement therapy [98]. The variability of hPSC models can also stem from the countless different protocols that are globally in use, leading to potential discrepancies in experimental findings. In addition, robust production and maintenance of clinical grade hPSC-derived cells such as cardiomyocytes can take upwards to several months. Even more challenging is the prospect of acquiring hPSCs from large cohorts of patients to generate consistent clinical data [100, 101]. Furthermore, hPSC-CMs are especially complicated since they are differentiated into an immature cellular state, which stems from the inherent lack of environmental cues that are critical for the initiation of physiological hypertrophy [102]. Compared to adult CMs, hPSC-CMs are significantly smaller in size and typically lack polyploidy, correct alignment, proper myofibril structure and an equivalent abundance of mitochondria. To facilitate the cellular maturation of hPSC-CMs, additional differentiation approaches such as 3D engineering of heart tissue combined with the application of mechanical stress and electrical stimulation must be pursued [103]. Lastly, if hPSC cultures are maintained over extensive periods of time, genetic instability may develop from chromosomal malformations and copy number variance [104–106]. Fortunately, a multitude of measures are currently available to either reduce or resolve the potential drawbacks of hPSCs [98]. Given the severity of the COVID-19 pandemic, the collective benefits and unique advantages of using hPSCs for modelling SARS-CoV-2 infection outweigh the limitations.

## Conclusions and Perspectives

COVID-19 has rapidly become a global pandemic resulting in millions of deaths and accumulation of different medical complications such as ARDS and myocardial injury [22, 27]. The swift transmission of SARS-CoV-2 is derived from viral aerosol particles that can quickly lead to infection if inhaled. Due to widespread expression of ACE2 and TMPRSS2 throughout several organ systems, the S glycoprotein is capable of infecting the lungs, heart, liver, kidneys, small intestine, pancreas, and brain [18, 54, 78]. In conjunction with potential activation of a cytokine storm, this degree of viral tropism often leads to the development of serious medical complications and enhanced disease progression in hospitalized patients [22, 27, 44]. The cardiovascular system is particularly of interest due to the bidirectional relationship between cardiovascular disorders and patients with severe SARS-CoV-2 infection. Cardiovascular disorders may enhance disease progression at the onset of SARS-CoV-2 infection or quickly arise from induction of the cytokine storm [17, 27, 47, 48]. While cardiovascular cells including cardiomyocytes, endothelial cells, vascular smooth muscle cells and pericytes express ACE2 [77, 107], the tropism of SARS-CoV-2 significantly differs in these cells based on in vitro experiments. Human PSC-CMs are permissive to SARS-CoV-2 infection and viral replication [92, 96], and PSC-derived blood vessel organoids can be infected by SARS-CoV-2 [108]. In contrast, endothelial cells are not infected by the virus [109, 110], though clinical data have indicated widespread damage in the endothelium in COVID-19 patients [111, 112]. In addition, SARS-CoV-2 is not detected in the myocardium of deceased COVID-19 patients [20], suggesting differential viral tropism between in vitro and in vivo. Therefore, the direct link between in vitro cell infection experiments and clinical manifestation seems to be obscure, leaving open questions for further investigation. Alternatively, cardiovascular damage in COVID-19 patients could be the secondary clinical outcome caused by the cytokine storm, which is triggered when SARS-CoV-2 directly infects the lungs. Although the overall mortality rate for SARS-CoV-2 is low, many recovered patients are still confronted with novel medical complications for months after infection. The long-term multi-organ consequences of SARS-CoV-2 infection demand the use of versatile and complementary models.

In order to gain a complete understanding of both the immediate and long-term effects of SARS-CoV-2 infection, hPSCs could serve as a versatile model for multiple-tissue infections [78]. Currently, hPSCs have already shown great promise in several SARS-CoV-2 studies focused on the lungs, heart, gut, and pancreas [78]. For example, in combination with in vivo and in silico models, hPSC-CMs have demonstrated significant value in elucidating the mechanisms of viral entry, cell death, and cytokine storm for indirect effects related to SARS-CoV-2 infection in neighboring cells and organs [40, 92, 95–97]. In addition, hPSCs exhibit powerful implications for SARS-CoV-2 clinical studies involving cell therapy or high throughput drug screening. When considering the limitations associated with hPSCs, technological advances in organoid development and CRISPR/Cas9 genome editing will likely nullify these drawbacks in the near future [98]. Although there are 15 approved vaccines with many more on the horizon, the global distribution and administration of these vaccines is a monumental task that will likely take upwards of months to years to accomplish [113–115]. As the COVID-19 pandemic continues to evolve every day with the discovery of new variants, it is vital that the most effective models are utilized to understand the biological and clinical mechanisms of SARS-CoV-2 infection.

## Data Availability

Not applicable.
